# Viral Metagenomics Reveals Diverse Viruses in the Feces Samples of Raccoon Dogs

**DOI:** 10.3389/fvets.2021.693564

**Published:** 2021-07-12

**Authors:** Shixing Yang, Yumin He, Xu Chen, Ullah Kalim, Yan Wang, Shuyu Yang, Haifeng Qi, HengZheng Cheng, Xiang Lu, Xiaochun Wang, Quan Shen, Wen Zhang

**Affiliations:** School of Medicine, Jiangsu University, Zhenjiang, China

**Keywords:** raccoon dogs, viral metagenomics, fecal virome, canine viruses, virus evolution

## Abstract

Raccoon dogs as an ancient species of *Canidae* are the host of many viruses, including rabies virus, canine distemper virus, severe acute respiratory syndrome coronavirus, and so on. With the development of raccoon dog breeding in recent years, some viruses which infected poultry or pigs were also detected from raccoon dogs. At present, the fecal virome of raccoon dogs has been rarely studied. Using an unbiased viral metagenomic approach, we investigated the fecal virome in raccoon dogs collected from one farm of Jilin Province, China. Many DNA or RNA viruses identified in those fecal samples were mainly from seven families, including *Circoviridae, Smacoviridae, Genomoviridae, Parvoviridae, Picornaviridae, Astroviridae*, and *Hepeviridae*. This study increased our understanding of the fecal virome in raccoon dog and provided valuable information for the monitoring, prevention, and treatment of viral diseases of these animals.

## Introduction

Raccoon dogs, also named *Nyctereutes procyonoides*, are members of the *Canidae* family. As endemic animals in East Asia, raccoon dogs are native to China, Japan, and other countries in Asia. In China, raccoon dogs, as one of the most important fur economic animals, are widely raised. About 10 million raccoon dog furs are gained for export abroad or domestic consumption each year. Viral disease is an important factor affecting the raccoon dog industry. In recent years, many viruses such as H5N1 influenza A virus (1), H9N2 influenza A virus (2), canine distemper virus (CDV) (3), canine parvovirus type 2 (4), amdoparvovirus (5), and porcine circovirus type 2 (6) were identified from farmed raccoon dogs of China. However, the viruses infecting raccoon dogs have not been studied systematically.

Viral metagenomics has proved to be a powerful tool for exploring new and existing viruses in a variety of tissue and fecal samples from humans and animals. Although the fecal virome has been investigated in canine using next-generation sequencing (7–9), limited fecal virome data is available for the raccoon dogs.

In this study, we investigated the viral composition in fecal samples from domestic raccoon dogs in Jilin Province of China using viral metagenomics. We reported previously uncharacterized raccoon dog viruses, including CRESS-DNA virus, Amdoparvovirus, Picornavirus, Cadicivirus, Astrovirus, Enterovirus, and viruses belonging to unclassified *Hepeviridae*. This study provided a baseline viral survey of raccoon dogs which will be helpful for future viral disease prevention and control in raccoon dogs.

## Materials and Methods

### Sample Collection and Preparation

Thirty fecal samples from healthy adult raccoon dogs were randomly collected from the same breeding farm (where about 100 raccoon dogs are raised) in Jilin Province, China, in 2014. All samples were collected by disposable materials and shipped on dry ice. About 1 g of each fecal sample was re-suspended in 2 ml phosphate-buffered saline, vigorously vortexed for 10 min, and then centrifuged at 12,000 rpm for 10 min. The fecal supernatant of each sample was transferred into a new 1.5-ml centrifuge tube and stored at −80°C for further use.

### Viral Nucleic Acid Extraction

Three sample supernatant pools were randomly generated, each of which contained 10 fecal supernatants. Then, 500 μl of each supernatant pool was filtered through a 0.45-μm filter (Merck Millipore, MA, USA) to remove bacterial and eukaryotic cell-sized particles and then treated with DNase (Turbo DNase from Ambion, Thermo Fisher, Waltham, MA, USA; Baseline-ZERO from Epicentre, Charlotte, USA, and Benzonase from Novagen, Darmstadt, Germany) and RNase (Promega, Madison, WI, USA) to digest unprotected nucleic acid at 37°C for 60 min (10). Viral RNA and DNA were extracted by using the QIAamp viral RNA Minikit (Qiagen, HQ, Germany).

### Library Construction and Bioinformatics Analysis

The cDNA of viral RNA was synthesized by using reverse transcription with six-base random primers, and then Klenow Fragment DNA polymerase (New England Biolabs, MA, USA) was used to generate the complementary chain of cDNA. Three libraries were constructed using the Nextera XT DNA Sample Preparation Kit (Illumina, CA, USA) and sequenced on the Miseq Illumina platform with 250-base paired ends with dual barcoding for each pool.

For bioinformatics analysis, the paired-end reads of 250 bp generated by MiSeq were debarcoded using vendor software from Illumina. An in-house analysis pipeline running on a 32-node Linux cluster was used to process the data. The reads were considered duplicates if bases 5 to 55 were identical and only one random copy was kept. Low sequencing quality tails were trimmed using Phred quality score 10 as the threshold. The adaptors were trimmed using the default parameters of VecScreen, which is NCBI BLASTn with specialized parameters designed for adaptor removal. The bacterial reads were subtracted by mapping to the bacterial nucleotide sequences from the BLAST NT database using Bowtie2 v2.2.4. The cleaned reads were *de novo* assembled by SOAPdenovo2 version r240 using Kmer size 63 with default settings (11). The assembled contigs, along with singlets, were aligned to an in-house viral proteome database using BLASTx (v.2.2.7), with an E-value cutoff of <10^−5^, where the virus BLASTx database was compiled using NCBI virus reference proteome (https://ftp.ncbi.nih.gov/refseq/release/viral/) to which viral protein sequences from NCBI nr fasta file (based on annotation taxonomy in the Virus Kingdom) were added. The candidate viral hits were then compared to an in-house non-virus non-redundant (NVNR) protein database to remove false positive viral hits, where the NVNR database was compiled using non-viral protein sequences extracted from NCBI nr fasta file (based on annotation taxonomy excluding the Virus Kingdom).

### Phylogenetic Analysis

Phylogenetic analyses were performed based on the predicted amino acid sequences in the present study, their closest viral relatives based on best BLASTx hits, and the representative members of related viral species or genera. Sequence alignment was performed using Clustal W with the default settings (12). The aligned sequences were trimmed to match the specific protein regions of the viral sequences obtained in the study. A phylogenetic tree with 500 bootstrap resamples of the alignment data sets was generated using the neighbor-joining method based on the *p*-distances model in MEGA-X (13). The bootstrap values (based on 500 replicates) for each node were given. The putative open reading frames (ORFs) in the genome were predicted by combining Geneious 11.1.2 software and NCBI ORF finder. The putative exon and intron were predicted by Netgenes2 at http://www.cbs.dtu.dk/services/NetGene2/.

### Nucleotide Sequence Accession Number

The genome and fragments of raccoon dog viruses obtained in this study were deposited in GenBank with the following accession numbers: MT457875–MT457884 and MT498597–MT498601. The raw sequence reads from the metagenomic library were deposited in the Shirt Read Archive of GenBank database under the following accession numbers: SRX9210517, SRX9210569, and SRX9210640.

## Results

### Viral Metagenomic Overview

**Figure 1:**
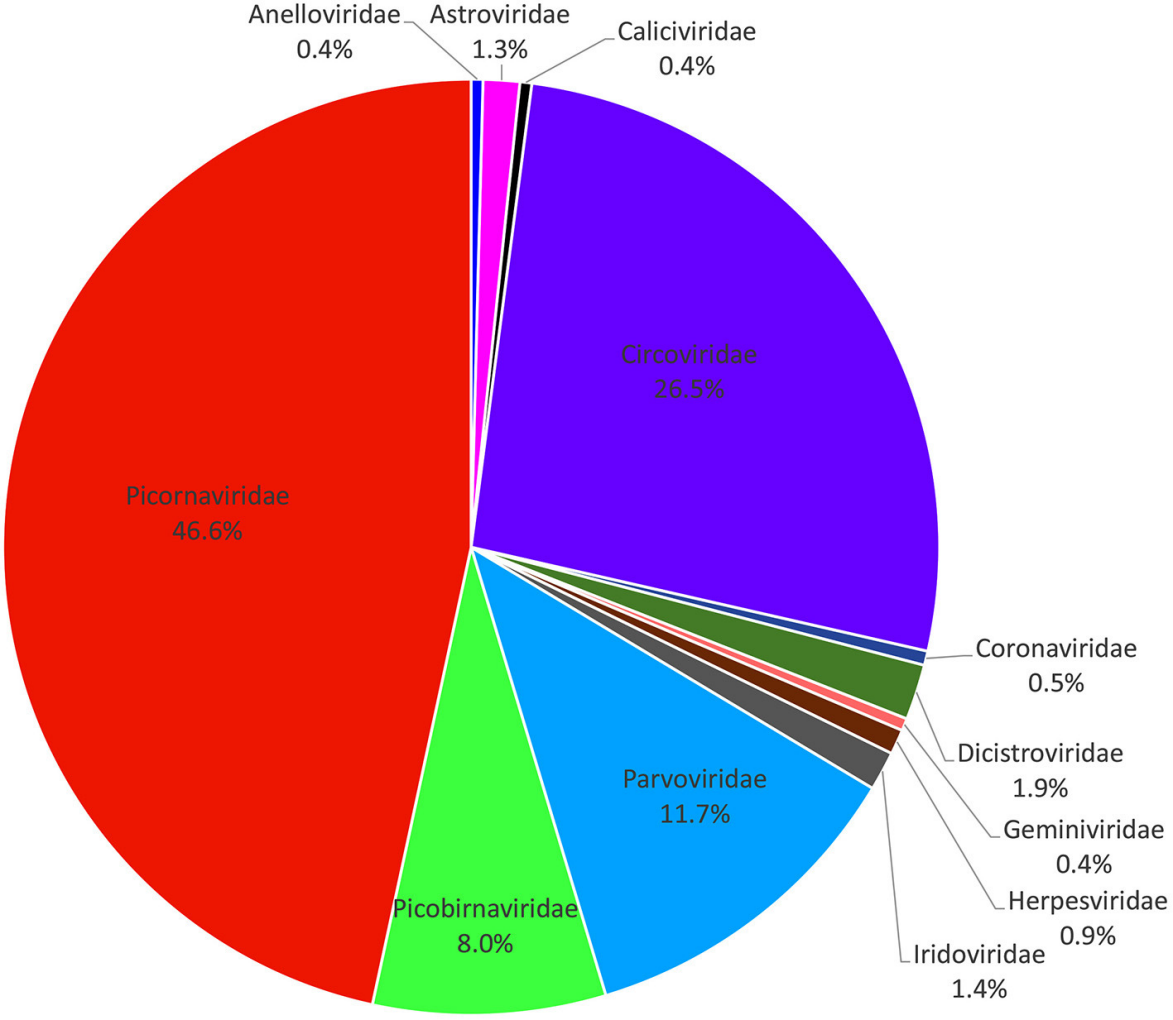
Composition of fecal virome detected in raccoon dogs. The percentage of virus sequences in different virus families was shown.

A total of 133,234 sequence reads (37,079 in library #1, 46,871 in library #2, and 49,284 in library #3) had the best matches with viral proteins (Supplementary Table 1). The putative mammalian viruses found in this study are shown in Supplementary Table 2. We analyzed the percentage of total sequence reads of different viral families in three libraries (a virus family with a total of sequence reads <40 was not statistically analyzed); the result is shown in Figure 1, where the most abundant animal virus family was *Picornaviridae* (46.62% of the total analytical animal virus reads), followed by the virus families of *Circoviridae* (26.49%), *Parvoviridae* (11.74%), *Picobirnaviridae* (8.03%), *Dicistroviridae* (1.92%), *Iridoviridae* (1.35%), *Astroviridae* (1.26%), *Herpesviridae* (0.86%), *Coronaviridae* (0.49%), *Geminiviridae* (0.43%), *Caliciviridae* (0.41%), and *Anelloviridae* (0.41%). The viruses including Iridovirus, Herpesvirus, Coronavirus, Calicivirus, and Anellovirus were omitted because of too few sequence reads to provide useful information. Those viruses with a large number of sequence reads belonging in the families of *Picornaviridae, Circoviridae, Parvoviridae, Dicistroviridae, Astroviridae*, and *Geminiviridae* were further fully characterized.

### A Virus Belonging to the Genus *Amdoparvovirus*

**Figure 2:**
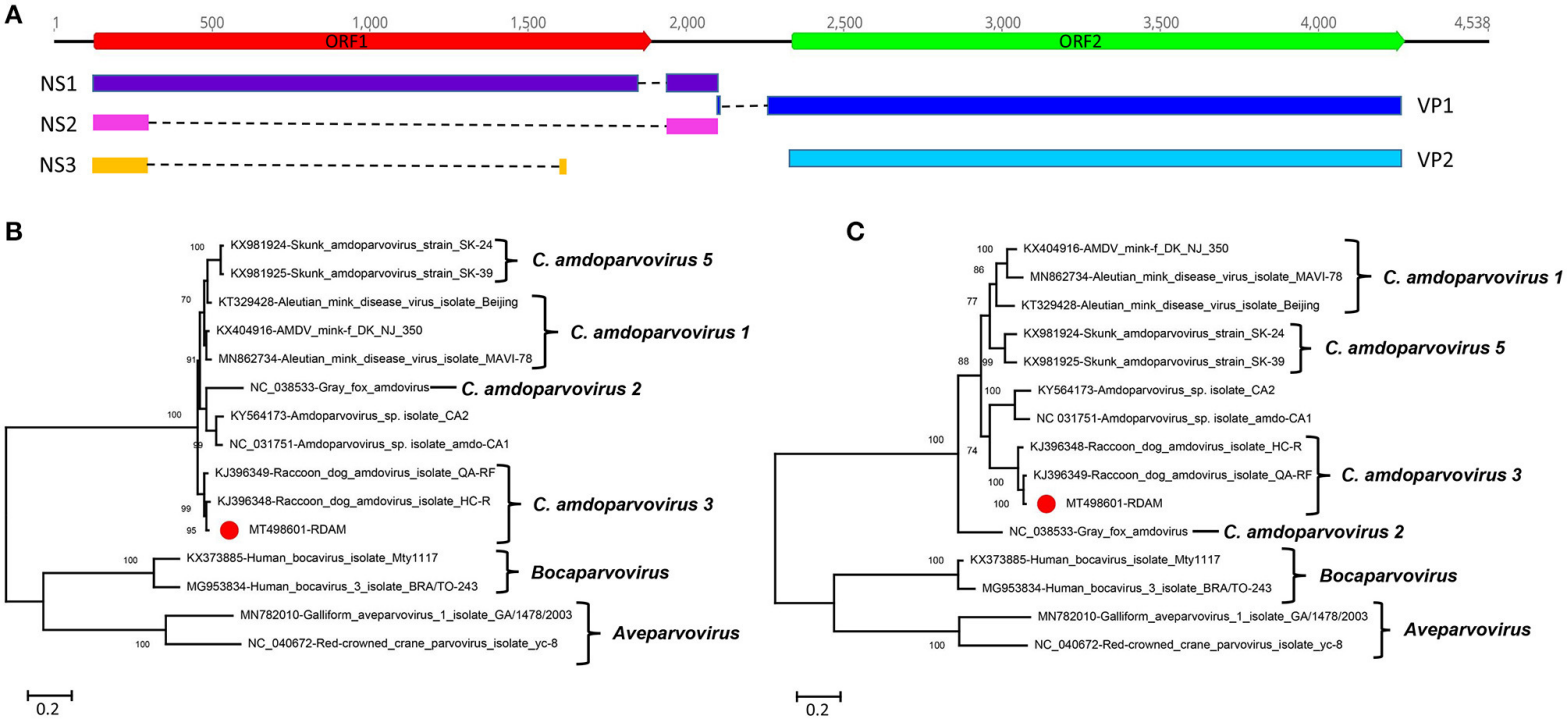
Genomic organization and phylogenetic analysis of the amdoparvovirus identified in raccoon dogs. **(A)** Genomic organization of RDAM. The open reading frames and viral encoding proteins of RDAM were marked with different colors. **(B)** Phylogenetic analysis based on the amino acid sequences of NS1 of RDAM and reference strains of the genus *Canine amdoparvovirus, Bocaparvovirus*, and *Aveparvovirus* in the family *Parvoviridae*. **(C)** Phylogenetic analysis based on the amino acid sequences of VP2 of RDAM and reference strains of the genus *Canine amdoparvovirus, Bocaparvovirus*, and *Aveparvovirus* in the family *Parvoviridae*. The RDAM identified in this study was marked with a red solid circle.

Here amdoparvovirus sequences were detected in all the three libraries, where one nearly complete genome of amdoparvovirus was obtained in library #2 using the Align/Assemble function in Geneious 11.1.2 and named RDAM. The genome of RDAM is 4,538 bp in length, which has two ORFs (ORF1 and ORF2) (Figure 2A). The length of ORF1 (128–1,891 nt) and ORF2 (2,336–4,273 nt) are 1,764 and 1,938 bp, respectively. Five viral proteins were predicted through comparing with previous amdoparvovirus sequences (GenBank no. KJ396349) and included three non-structural proteins—NS1, NS2, and NS3—and two structural proteins—VP1 and VP2. All those five proteins were encoded through different splicing patterns: NS1 of 641 aa (amino acid) (128–1,883 nt spliced to 1,973–2,142 nt), NS2 of 114 aa (128–306 nt spliced to 1,973–2,138 nt), NS3 of 66 aa (128–306 nt spliced to 1,659–1,680 nt), VP1 of 688 aa (2,135–2,144 nt spliced to 2,217–4,273 nt), and VP2 of 645 aa (2,336–4,273 nt). Four helicase motifs were present at the C-terminal of RDAM NS1, which showed a high conservation among all four of the *Amdoparvovirus* species (Supplementary Figure 1) (14). The Walker motif A and B′ of RADM is identical with *Carnivore amdoparvovirus 2*, the Walker motif B of RADM has one aa different from *Carnivore amdoparvovirus 2*, while the Walker motif C of RADM is the same with *Carnivore amdoparvovirus 1* and *Carnivore amdoparvovirus 5*. RADM has a 14-Gly aa conservative motif at the N-terminal of VP2 just like other *Carnivore amdoparvovirus 3* (GenBank nos. KJ396349 and KJ396348) (data not shown).

Two phylogenetic trees were constructed based on the protein sequences of NS1 and VP2 and reference sequences, including four *Amdoparvovirus* species (missing *Carnivore amdoparvovirus 4* because it has no complete genome) and the two genera of *Aveparvovirus* and *Bocaparvovirus* as outgroups (Figures 2B,C). The result showed that four *Amdoparvovirus* species were clearly delineated in the NS1 and VP2 phylogenetic trees, but a clade that consisted of two viruses (KY564173 and NC_031751) was different in those two phylogenetic trees. The RADM that clustered with two *Carnivore amdoparvovirus 3* reference viruses formed a clade. A sequence analysis using BLASTp in NCBI showed that the NS1 of RADM shared the highest aa sequence identity (97.97%) with the strain QA-RF (GenBank no. KJ396349), while the VP2 of RADM had the highest aa sequence identity (97.52%) with the strain HC-R (GenBank no. KJ396348). Both of the referenced strains were identified from raccoon dog tissue samples that were collected in Jinlin Province of China in 2013 during an outbreak of a disease in farms (5). We found only 12 and 16 aa sequence variations in the NS1 and VP2 region of RADM by comparing with the strain QA-RF and HC-R, respectively.

Finally, pairwise sequence identities within and between each species were analyzed for NS1 and VP2 proteins (Supplementary Figure 2). The RADM NS1 shared 64.7–98.0% aa sequence identity with other reported viruses, while the RADM VP2 showed 76.0–97.5% aa sequence identity with the other strains. The ICTV classification criteria of species in the family *Parvoviridae* state that viruses within a species generally encode NS1 proteins that show >85% aa sequence identity; therefore, the RADM belongs to the same genus as *Carnivore amdoparvovirus 3*.

### A Virus Belonging to the Genus *Dicipivirus* Firstly Identified in a Raccoon Dog

**Figure 3:**
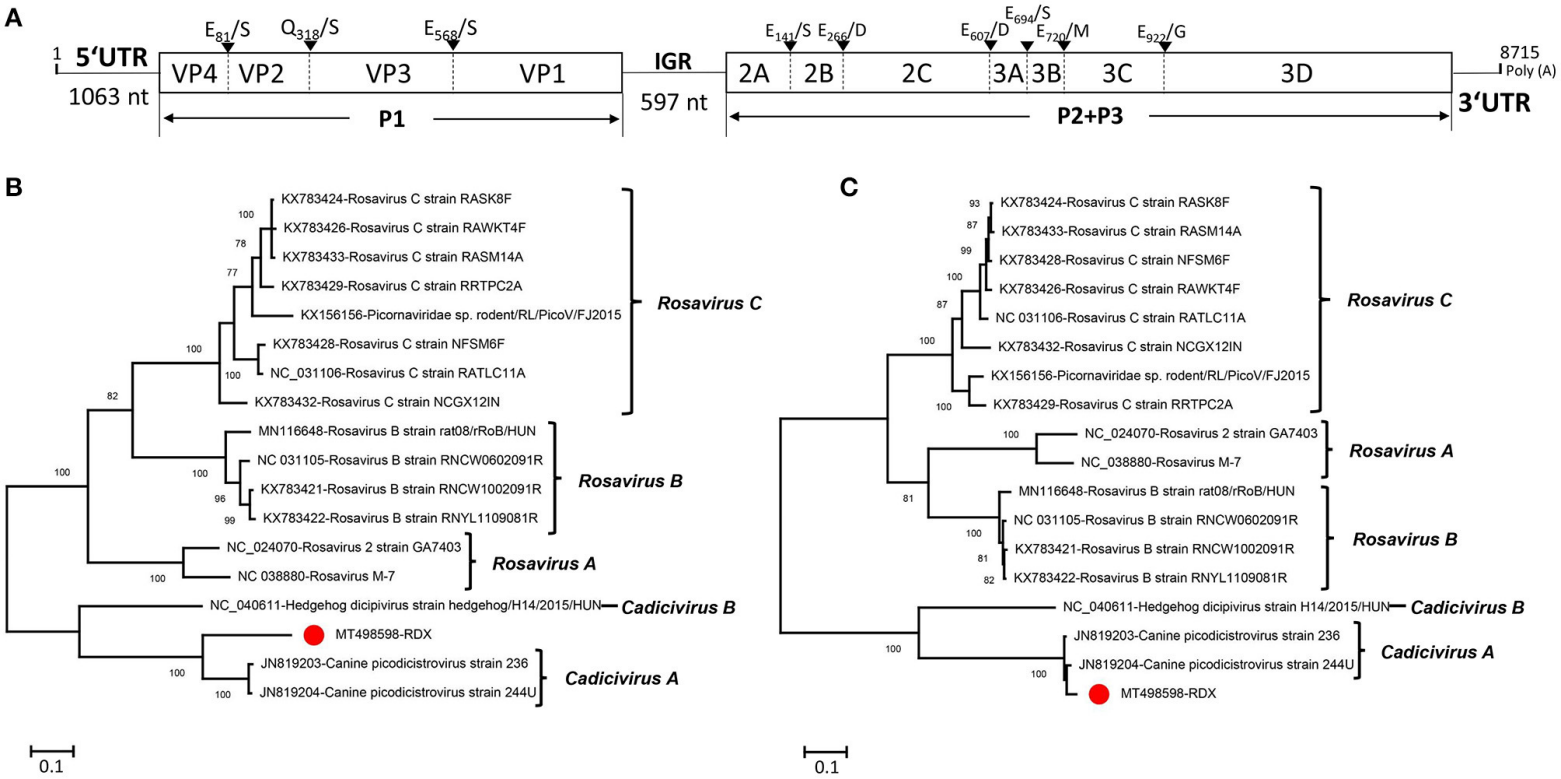
Genomic organization and phylogenetic analysis of the novel dicipivirus identified in raccoon dogs. **(A)** Genomic organization and predicted protein cleavage sites of RDX. The open reading frames and viral encoding proteins of RDX were drawn. The protein cleavage sites of the RDX strain was obtained through comparing to canine picodicistrovirus (JN819204). IGR, intergenic region; nt, nucleotide; UTR, untranslated region. **(B)** Phylogenetic analysis based on the amino acid sequences of P1 of RDX and reference strains of the genus *Cadicivirus* and *Rosavirus* in the family *Picornaviridae*. **(C)** Phylogenetic analysis based on the amino acid sequences of P2+P3 of RDX and reference strains of the genus *Cadicivirus* and *Rosavirus* in the family *Picornaviridae*. The RDX identified in this study was marked with a red solid circle.

In this study, dicipivirus sequences were detected in all three libraries (461 reads in library #1, 3,234 reads in library #2, and 153 reads in library #3), among which one nearly complete genome of dicipivirus was assembled from library #2 and named RDX. The nearly complete genome of the RDX is 8,715 nt in length, including a 1,063-nt 5′ end sequence, a 597-nt intergenic region (IGR), and a 188-nt 3′ end sequence (Figure 3A). The length of IGR in RDX is nine nt longer than the corresponding IGR of canine picodicistrovirus. The nucleotide identity of IGR between RDX and canine picodicistrovirus (JN819204) is 94.56%. The genome of RDX has two ORFs (ORF1 and ORF2), the same with other dicipiviruses; the P1 encodes a viral capsid polyprotein of 882 aa which was spliced into four capsid proteins VP4 (81 aa), VP2 (237 aa), VP3 (250 aa), and VP1 (314 aa). The potent cleavage sites between VP4/2, VP2/3, and VP3/1 are E_81_/S, Q_318_/S, and E_568_/S, respectively (Figure 3A). Comparing with the P1 of reference virus JN819204, the VP4 protein of RDX is 37 aa longer than JN819204, the VP2 of RDX is one aa shorter than JN819204, the length of RDX VP3 is the same with JN819204, and the VP1 of RDX is two aa longer than JN819204. These proteins had 84.09, 73.95, 72.98, and 68.49% aa identity to JN819204, respectively. The P2/P3 encodes a viral non-structural protein polycursor of 1,405 aa, which is one aa shorter than the corresponding P2/P3 of JN819204. The P2 region encodes three non-structural proteins, including 2A, 2B, and 2C. The potent cleavage sites between 2A/2B, 2B/2C, and 2C/3A are E_141_/S, E_266_/D, and E_607_/D, respectively (Figure 3A). Being similar to canine picodicistrovirus, the 2A of RDX does not contain the conservative catalytic aa residues of chymotrypsin-like proteases such as the NPGP or H-box/NC motifs. The 2C of RDX possesses the NTP binding motif GXXGXGKS (G_404_ KPGCGKS) and the putative helicase motif D_455_DLGQ. Comparing with the P2 of JN819204, 2B of RDX has one aa deletion. The P2 of RDX shares 94.41% aa identity with JN819204. The P3 region in the genomes of RDX encodes 3A, 3B (viral protein genome-linked, also named VPg), 3C^pro^ (protease), and 3D (RNA-dependent RNA polymerase) proteins; the potent cleavage sites between 3A/3B, 3B/3C, and 3C/3D are E_694_/S, E_720_/M, and E_922_/G, respectively (Figure 3A). The lengths of 3A, 3B, 3C, and 3D of RDX are the same with those of JN819204. Similar to JN819204, the 3C^pro^ of RDX contains the conserved GXCG motif (G_878_FCG), which is part of the active site of the protease, while the catalytic triad of 3C^pro^ in RDX is not the His-Glu-Cys but His_866_-Thr_875_-Cys_880_. The conserved GXH motif of JN819204 functioning as part of the substrate binding pocket of the protease was not found in 3C^pro^ of RDX. 3D of RDX contains the conserved K_1086_DELR, Y_1257_ GDD, and F_1306_LKR, although the GGXPSG motif is replaced by the G_1215_AMPSG motif in RDX, which is similar to that of JN819204. The P3 of RDX had 96.36% aa identity to JN819204.

Two phylogenetic trees were drawn based on the aa sequences of P1 and P2/P3 regions that included the reference strains of two *Dicipivirus* species and the three *Rosavirus* species as outgroups. The result showed that RDX clustered with two canine picodicistroviruses (JN819203 and JN819204) and formed a clade (Figures 3B,C). Our data suggested that RDX belonged to the species of *Cadicivirus* A in genus *Dicipivirus* based on the result of a phylogenetic analysis and a homology comparison. To our best knowledge, it is the first time that dicipivirus was detected in the fecal samples of raccoon dogs.

### An Enterovirus H Virus

In this study, an incomplete enterovirus genome was determined in library #3 and named RDEN. The incomplete genome of enterovirus strain RDEN is 5,444 nt in length, including 625 nt of 5′ terminal sequences, complete P1 and P2 regions, and partial P3. The P1 of RDEN encoded viral capsid polyprotein of 872 aa which was cleaved into four capsid proteins, including VP4 (69 aa), VP2 (251 aa), VP3 (244 aa), and VP1 (308 aa). The potent cleavage sites between VP4/2, VP2/3, and VP3/1 are separately K_69_S, Q_320_G, Q_564_A, and T_872_L, respectively. These proteins (VP4, VP2, VP3, and VP1) shared 63.77, 62.15, 56.33, and 43.94% aa identity to the simian enterovirus SV4 (NC_038309), respectively. The P2 of RDEN is 575 aa in length and was cut into three non-structural proteins, including 2A (148 aa), 2B (99 aa), and 2C (328 aa), respectively. The putative cleavage sites of A/2B, 2B/2C, and 2C/3A are Q_1020_G, Q_1119_S, and Q1447G, respectively. The 2C of RDEN had the NTP binding motif GXXGXGKS (G_1248_SPGTGKS). The P2 of RDX had 70.14% aa identity to enterovirus D94 (DQ916376).

**Figure 4:**
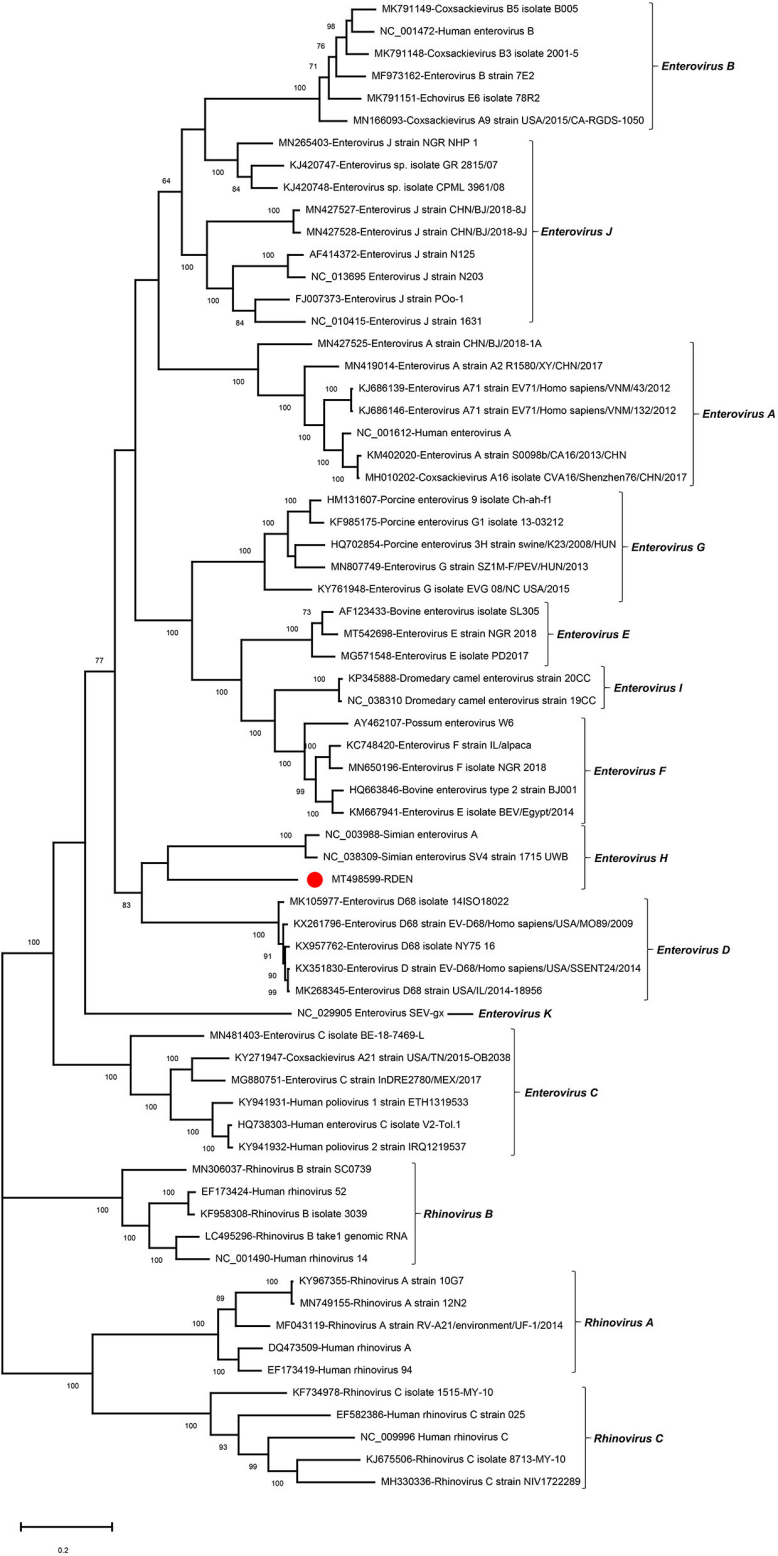
Phylogenetic analysis based on the amino acid sequences of P1+P2 of RDEN and reference strains of the genus *Enterovirus* in the family *Picornaviridae*. The RDEN identified in this study was marked with a red solid circle.

To investigate the genetic relationship of RDEN with other enteroviruses, the phylogenetic trees were constructed based on P1+P2 region, including the reference strains of 11 *Enterovirus* species and three *Rhinovirus* species as outgroups. The result indicated that the RDEN clustered with two *Enterovirus H* strains (NC_003988 and NC_038309), forming an independent clade which suggested that EDEN belonged to a member in the species of *Enterovirus H* (Figure 4). Brown *et al*. suggested that the nucleotide identity in the “gray zone” of 70–75% VP1 nt, a more stringent value of 88% VP1 aa identity, is more appropriate for routine typing (15). The VP1-coding sequence of RDEN showed 44.3% nt and 43.94% aa identity to the closest relative, a simian enterovirus SV4 (NC_038309), suggesting that RDEN belonged to a new type of *Enterovirus H*. To our best knowledge, it is the first time that enteroviruses were detected in fecal samples of raccoon dogs.

### CRESS-DNA Viruses

**Figure 5:**
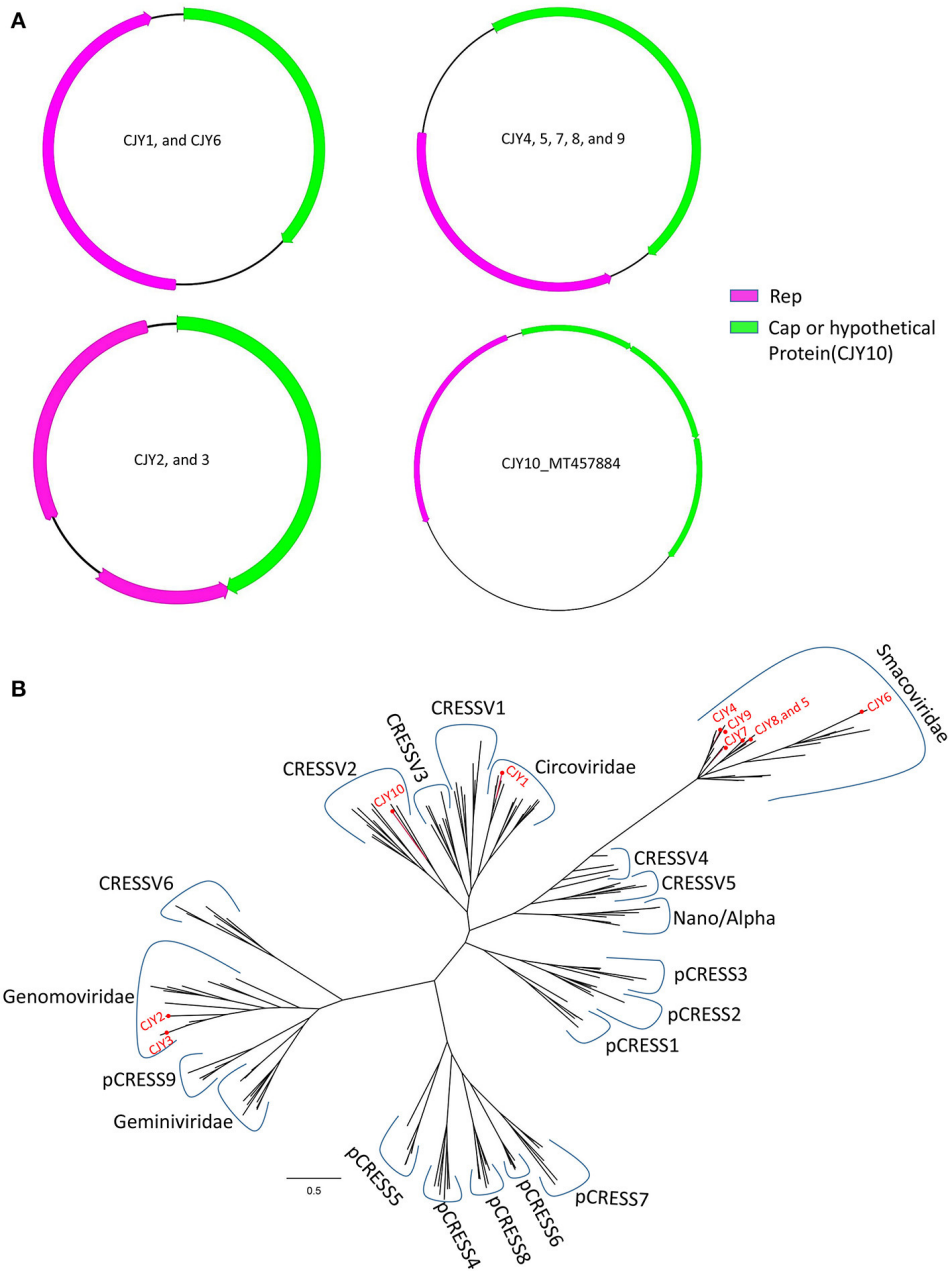
Genomic organization and phylogenetic analysis of 10 CRESS-DNA viruses identified in raccoon dogs. **(A)** Genomic organization of CJY1-10. The Rep protein and Cap protein of CJY1-10 were separately marked with pink and green. The arrow indicated the direction of gene coding. **(B)** Phylogenetic analysis based on the amino acid sequences of Rep of CJY1-10 and reference strains of CRESS-DNA virus, unclassified CRESS-DNA virus (CRESSV1-6), and bacterial plasmids (pCRESS1-9). The CJY1-10 identified in this study was marked with a red solid circle.

In this study, 10 complete genomes of CRESS-DNA virus were obtained and named CJY1–CJY10, respectively. Nine genomes of the 10 CRESS-DNA viruses (CJY2 and CJY4–6 from library #1; CJY1 and CJY7 from library #2; and CJY3, CJY8, and CJY9 from library #3) ranged from 2,030 to 2,610 bp in length, but CJY10 (from library #2) had a larger genome of 3,975 bp in length. Nine of those virus genomes except for CJY10 had two ORFs encoding Rep and Cap proteins, while the genome of CJY10 has four ORFs encoding one Rep and three hypothetical proteins. As shown in Figure 5A, there are four types of gene structures in those virus genomes. Seven CRESS-DNA viruses (CJY2–5 and CJY7–9) contained two bidirectional ORFs, where the encoding Rep gene of CJY2 and CJY3 contains an intron. The encoding ORFs of CJY1 and CJY6 are in the same direction, while in the four encoding ORFs of CJY10 the Rep protein is in the opposite direction to the remaining three hypothetical proteins.

A BLASTp search in the GenBank based on the aa sequences of Rep showed that CJY1 shared the highest identity of 63.07% with a giardia-associated CRESS-DNA virus isolate 84-AMS-01 (MT293425), CJY2 shared the highest identity of 96.32% to a chicken genomovirus isolate mg2_75 (MN379601), CJY3 shared the highest identity of 87.84% to a gila monster-associated gemycircularvirus isolate gila_283 (MH378453), CJY4 shared the highest identity of 79.01% to a porcine-associated porprismacovirus isolate 17489x27_1438 (MH111126), CJY5 shared the highest identity of 71.94% to a porcine-associated porprismacovirus 6 isolate XP1 (NC_024776), CJY6 shared the highest identity of 94.98% to a chicken virus isolate mg7_59 (MN379596), CJY7 shared the highest identity of 64.71% to a capybara associated smacovirus isolate 1_cap1_104 (MK570200), CJY8 shared the highest identity of 90.94% to a rat stool-associated circular ssDNA virus isolate Mu/10/1799 (KP860907), CJY9 shared the highest identity of 79.01% to a porcine-associated porprismacovirus isolate 17489x27_1438 (MH111126), and CJY10 shared the highest identity of 48.06% to a circovirus species isolate PoCirV_VIRES_GX05_C2 (MK377556), respectively.

For phylogenetic analysis, 175 Rep proteins including the reference of CRESS-DNA virus, unclassified CRESS-DNA virus (CRESSV1-6), bacterial plasmids (pCRESS1-9), and 10 CRESS-DNA viruses from this study as well as those that have the best matches with them were used for alignment and construction of the phylogenetic tree. As shown in Figure 5B, CJY4–9 fell into the same clade of *Smacoviridae*, CJY2 and CJY3 together with other genomoviruses formed a clade, CJY1 clustered with circoviruses, while the remaining CJY10 belonged to the cluster of CRESSV2.

### Other Viruses That Only Obtained Short Fragments

Some other viruses including one astrovirus belonged to the genus *Mamastrovirus*, and one hepe-like virus was identified in library #2. Their full genomes were not obtained using the method of assembling or PCR amplification because of a limited number of viral sequences in the sequencing data or low titers of virus in the samples. A phylogenetic analysis was done based on the short fragments obtained.

**Figure 6:**
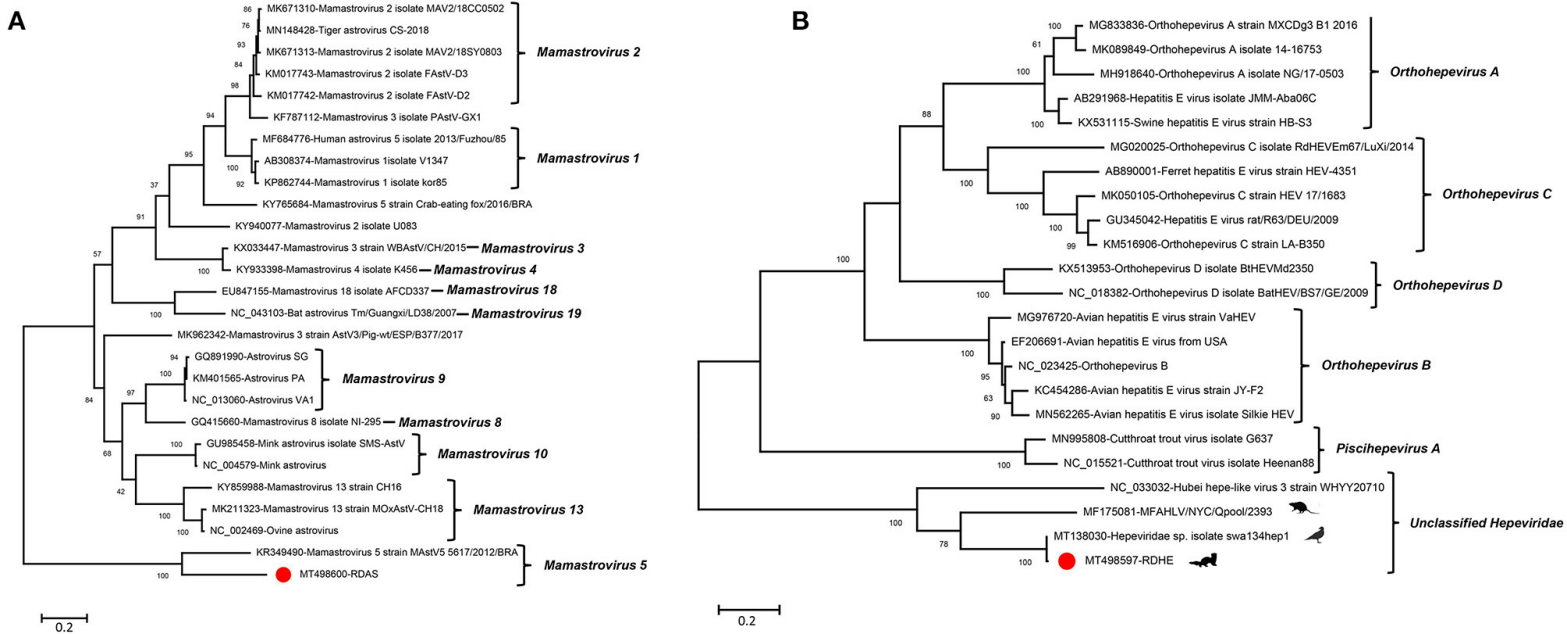
Phylogenetic analysis of an astrovirus and a hepe-like virus identified in this study. **(A)** For astrovirus (RDAS). **(B)** For hepe-like virus (RDHE). The two viruses identified in this study were marked with a red solid circle.

An incomplete astrovirus genome of 2,351 nt in length, including partial ORF1a and ORF1b, was assembled in library #2 and was named RDAS. The partial ORF1a encodes 346 aa and shares 53.0% aa identity with Mamastrovirus 5 strain MastV5_5617/2012/BRA (KR349490) which was isolated from the fecal specimens of dogs with diarrhea. The partial ORF1b encoded 432 aa and shared 73.9% aa identity to Mamastrovirus 5 strain MastV5_5617/2012/BRA (KR349490). The phylogenetic trees were constructed based on the aa sequences of partial ORF1b that included RDAS and 11 reference viruses belonging to genus *Mamastrovirus*. The result showed that RDAS clustered with MastV5_5617/2012 and formed an independent clade (Figure 6A). This is the first time that astroviruses have been identified in fecal samples from raccoon dogs. The current classification of *Mamastrovirus* species is based on the hosts in which they were found. We therefore suggested that RDAS belonged to a new species in the genus of *Mamastrovirus*.

One novel hepe-like virus was identified in this study and named RDHE. The incomplete genome of RDHE was 2,464 nt in length, which encoded a partial non-structural protein. It shares the highest aa identity of 99.15% to *Hepeviridae* sp. isolate swa134hep1 (MT138030) which was isolated from anal swabs of wild birds. To investigate the genetic relationship of RDHE with other hepeviruses, a phylogenetic tree was constructed based on the partial non-structural protein of RDHE, the reference strains of *Orthohepevirus* A, *Orthohepevirus* B, *Orthohepevirus* C, *Orthohepevirus* D, *Piscihepevirus*, and unclassified hepeviruses. The result indicated that RDHE clustered with three other unclassified hepeviruses, forming an independent clade at a far distance from other classified hepeviruses (Figure 6B). Those hepeviruses in the same clade with RDHE were identified from different living organisms, including house centipedes, mice, and wild birds.

## Discussion

Viral metagenomics has recently been used in numerous animal virus discoveries, providing information on the composition of animal viromes and helping to identify zoonotic and emerging viruses. We described here the viral communities in the fecal samples of raccoon dogs and found many new and previously known viruses through unbiased high-throughput sequencing analysis. The wild raccoon dog is an important vector of viral diseases, such as rabies and canine distemper. Unsurprisingly, no rabies virus and canine distemper virus were detected in this study, which was likely because the raccoon dogs are fed in intensive culture, and standardized vaccination procedures are adopted in the breeding process.

Feeding environment and food-borne safety are effective means for preventing raccoon dogs from being infected with viral diseases. However, at present, the breeding mode of raccoon dogs in China is mainly small-scale family feeding and mixed feeding which lack unified, effective, and standardized management systems and safe food sources. Thus, the risk of cross-species transmission of virus to raccoon dogs may be increased. In recent years, various viruses detected from farmed raccoon dogs have seriously threaten this industry. Some viruses are transmitted to raccoon dogs through food-borne transmission, for example, raccoon dogs may get infected with H5N1 and H9N2 avian influenza viruses by eating contaminated poultry or wild birds (1, 2). Some viruses infect raccoon dogs through environmental pollution. For example, it has been recently reported that raccoon dogs have been infected with porcine circovirus type 2 from nearby pig farms, resulting in reproductive failure (6).

Hepeviruses are small, non-enveloped +ssRNA viruses with a genome of 6.4–7.2 kb in length. The genome of a hepevirus consists of three partially overlapping ORFs. ORF1 encodes viral non-structural proteins, ORF2 encodes a viral capsid protein, while ORF3 encodes a small immunoreactive protein which has multiple functions associated with virion morphogenesis, egress, and viral pathogenesis. The family *Hepeviridae* is comprised of two genera and five species. Members of the genus *Piscihepevirus* only infect trout, while members of the genus *Orthohepevirus* infect mammals and birds. Hepatitis E virus (HEV) belongs to *Orthohepevirus* A, which is responsible for self-limited acute hepatitis in humans and several mammalian species (16). The species *Orthohepevirus* B includes avian HEV that causes hepatitis–splenomegaly syndrome in chickens (17). Some unclassified hepeviruses were recently detected from various samples of animals, insects, or birds (18, 19). This indicates that there may be more hepeviruses in nature than we currently know. An unclassified hepevirus RDHE detected in this study had the highest homology (99.15%) with the unclassified hepatitis virus detected in the anal swab of wild birds, which might be caused by the daily diet of raccoon dogs.

CRESS-DNA viruses are a large group of viruses characterized as small, circular ssDNA genomes encoding a replication-associated protein (Rep). It has been reported that six CRESS-DNA virus families were detected in eukaryotic organisms including *Bacilladnaviridae, Circoviridae, Geminiviridae, Genomoviridae, Nanoviridae*, and *Smacoviridae*. Among them, the families *Geminiviridae* and *Nanoviridae* were mainly reported in plants (20), the family *Bacilladnaviridae* was identified from different diatoms (21), members of the *Circoviridae* were found in numerous vertebrate and invertebrate organisms (22), and the families *Genomoviridae* and *Smacoviridae* without definitive hosts were detected in various biological samples (23). In this study, a total of 10 CRESS-DNA viruses were detected. The aa analysis showed that CJY2-10 had the highest homology with those strains which were isolated from chicken, pig, rat, lizard, and capybara separately. CJY1 had the highest homology with a strain isolated from *Giardia*. The foundation of these strains might have originated from parasite infection or food consumption. We cannot rule out that raccoon dogs are the natural hosts of those CRESS-DNA viruses because CRESS-DNA viruses have been identified from various animals including canine.

Amdoparvoviruses, members of the genus *Amdoparvovirus* from the family *Parvoviruidae*, have five different species, including *Carnivore amdoparvovirus 1, Carnivore amdoparvovirus 2, Carnivore amdoparvovirus 3, Carnivore amdoparvovirus 4*, and *Carnivore amdoparvovirus 5*. Amdoparvoviruses are small, round, non-enveloped -ssDNA viruses with a genome of about 4.8 kb in length, which encodes three non-structural proteins (NS1, NS2, and NS3) and two structural proteins (VP1 and VP2) through alternative mRNA splicing (24). Amdoparvoviruses were firstly identified in the 1950s in minks and caused Aleutian disease (25). In addition to minks, amdoparvovirus can also infect other mustelids and small carnivores including ferrets, badgers, weasels, otters, raccoons, striped skunks, bobcat, dog, cat, arctic foxes, red pandas, and so on (26, 27). An amdoparvovirus (named RDAM) was detected in fecal samples of raccoon dogs in the present study. The aa homology of RDAM detected in this study was close to that of the strain QA-RF which caused infant fox and raccoon dog disease as reported in 2014 in Jilin of China (5). Although the raccoon dogs sampled in this study did not show clinical symptoms, it was not enough to draw the conclusion that RDAM was not pathogenic, and the virus may be in the incubation period at the time of sampling or only causes subclinical symptoms in adult raccoon dogs. Compared with QA-RF, both the NS and VP regions of RDAM had aa mutations, so it was not ruled out that the virulence of the virus decreased after adaptive evolution and did not cause obvious infection diseases. Further animal experiments are needed to confirm whether it is pathogenic. Raccoon dogs infected with the admoparvovirus QA-RF strain showed obvious clinical symptoms including anorexia, emaciation, growth retardation, thirst, chronic diarrhea, and unkempt fur; necropsy often revealed cyanosed splenomegaly, enlargement of mesenteric lymph nodes, and renal cortex congestion and brittleness. The rate of illness was 4–8%, while the death rate was about 60% before the age of 4 months. At present, no effective vaccine is available for protecting raccoon dogs from admoparvovirus infection. The health status of those farmed raccoon dogs in this study needs to be closely monitored to avoid the spread of this disease.

Picornaviruses are small, round +ssRNA viruses with a genome range of 6.7–9.9 kb in length. At present, the family *Picornaviridae* includes 63 genera and 147 species. Most of the picornaviruses have a similar genome structure which encodes a single polyprotein transcribed from a single ORF flanked by 5′ and 3′ untranslated regions (UTR) and a poly (A)-tail, while a special genus, *Dicipivirus*, has two ORFs that were separated by an IGR carrying an IRES. The dicipivirus was firstly identified from the fecal samples of dogs in 2012 (28); recently, another dicipivirus was detected from the fecal and tissue samples of northern white-breasted hedgehogs in 2018 (29). Dicipivirus was detected in raccoon dogs for the first time in this study. Based on the aa analysis of P2 and P3 regions, the strain RDX in this study had the highest homology (>94%) to strain 224U (GenBank no. JN819204) isolated from dog feces in Hong Kong in 2009, while the protein encoded in P1 region (VP1–VP4) was quite different, and the identity between the two viruses of VP1–VP4 ranged from 68.5 to 84.09%. The aa sequence difference based on P1 and P2–P3 regions implied that recombination may have occurred in the genome. The recombination analysis is difficult to be conducted because there are only three related genomes of *Cadicivirus A* available in GenBank at present. The P1 region encodes the capsid protein of the virus, which functions to recognize the host receptor (30). The difference in capsid protein between two strains of *Cadicivirus A* isolated from different species may indicate that the virus evolved to recognize different receptors of different species (31). IRES has been proven to have a function in viral pathogenesis. Almost all picornaviruses possess only one IRES element at the 5′ UTR upstream from VP4. Differently from other members in *Picornaviridae* family, dicipiviruses have two IRESs, one is at the 5′ UTR upstream from VP4, and the other one is located between the P1 and P2 regions. Both IRES elements were confirmed to be functional by DNA transfection and RNA transfection studies. The nucleotide homology of RDX in the second IRES region had 94.56% identity with that of the strain 224U. The high conservation of this region may be helpful for its function as IRES (32). Previous epidemiological investigations showed that 47 out of 368 dog fecal samples (12.8%) were positive in dicipivirus detection (28). Although no epidemiological investigation of RDX was carried out in this study, dicipivirus sequences were detected in all three virus libraries. Whether this virus may potentially cause an infection still requires more epidemiological data and animal experiments.

Enteroviruses (EVs) are members of the genus *Enterovirus* in the family *Picornaviridae* and are currently divided into 15 species, including *Enterovirus A–L* and *Rhinovirus A–C*, based on sequence diversity (33). Enteroviruses can infect multiple mammals, including humans, non-human primates, pigs, sheep, cattle, and camels (33). Humans who are infected with EV present with different clinical symptoms ranging from mild febrile illness to severe forms, while animals that are infected with EV are usually asymptomatic. EVs are small, non-enveloped +ssRNA viruses with an icosahedral capsid. The length of an EV genome is about 7.5 kb, having a poly-adenylated 3′ end, while a small protein VPg covalently links to the 5′ terminus. The single ORF of EVs is flanked by two untranslated regions (5′ UTR and 3′ UTR), which can be translated into a polyprotein and further cleaved into four structural proteins (VP1–VP4) and seven non-structural proteins (2A, 2B, 2C, 3A, 3B, 3C, and 3D). At present, only two strains of *Enterovirus H* have been isolated from simians and humans with viral hepatitis. In this study, an *Enterovirus H* (named RDEN) was detected in raccoon dog feces for the first time. The RDEN had the highest capsid protein identity with the strain 1715 UWB (43.94–63.77%). Animals infected with enterovirus are usually asymptomatic. The RDEN observed in this study also came from healthy raccoon dogs, which suggested that they may not be pathogenic.

In addition, astroviruses were also found in feces of raccoon dogs. Astroviruses are small, non-enveloped +ssRNA viruses with a genome of 6.8–7 kb in size. The 5′ terminus is linked to a VPg protein and the 3′ end has a poly (A) tract. The genome of astrovirus has three ORFs, including ORF1a, ORF1b, and ORF2. ORF1a and ORF1b encode the viral protease and polymerase separately, while ORF2 mainly encodes a VP90 capsid precursor protein. The family *Astroviridae* includes two genera, *Avastrovirus* (three species) and *Mamastrovirus* (19 species). Members of the genus *Avastrovirus* infect birds and cause intestinal or extra-intestinal manifestations (34), while members of the genus *Mamastrovirus* mainly infect mammals such as humans, felines, pigs, bovines, sheep, and minks and cause gastroenteritis (35, 36). Due to the low abundance of virus in the libraries, the complete genome of RDAS could not be obtained. A phylogenetic analysis showed that the RDAS was clustered with a dog fecal isolate strain, which indicated that the RDAS was probably carried by raccoon dogs themselves. Canine astrovirus has been considered the primary cause of gastroenteritis in young animals worldwide. Whether RDAS can cause raccoon dog disease needs to be further closely followed up.

In conclusion, this study provided an overview of the fecal virome of raccoon dogs and significantly increased our understanding of the viral diversity found in samples from animals in family *Canidae*. This study ultimately provides useful information for monitoring the health of these animals and may aid in the prevention and treatment of viral diseases in raccoon dogs.

## Data Availability Statement

The datasets presented in this study can be found in online repositories. The names of the repository/repositories and accession number(s) can be found in the article/Supplementary Material.

## Ethics Statement

The animal study was reviewed and approved by Jiangsu University Ethics Committee on the use of animals and complied with Chinese ethics laws and regulations. Written informed consent was obtained from the owners for the participation of their animals in this study.

## Author Contributions

ShiY and WZ conceived and designed the study and contributed to writing of the manuscript. ShiY, YH, XC, and UK carried out the study. YW, ShuY, HQ, HC, XL, XW, and QS analyzed the data. All the authors read and approved the final manuscript.

## Conflict of Interest

The authors declare that the research was conducted in the absence of any commercial or financial relationships that could be construed as a potential conflict of interest.

## Supplementary Material

Supplementary Figure 1 Comparison of amino acid sequences of helicase motifs in members of the genus *Amdoparvovirus*. A sequence logo for each species was generated with Weblogo 3. Four walker domains were marked with square boxes.

Supplementary Figure 2 Pairwise comparison of RADM with the reference strains of *Canine amdoparvoviruses*. **(A)** Based on the NS1. **(B)** Based on the VP2. The percent identity between RADM and other strains was marked with red.

Supplementary Table 1 The Summary of library information of raccoon dogs.

Supplementary Table 2 The Sequence numbers of animal virus in different libraries of this study.

## References

[1] Qi X Li X Rider P Fan W Gu H Xu L. Molecular characterization of highly pathogenic H5N1 avian influenza A viruses isolated from raccoon dogs in China. PLoS ONE. (2009) 4:e4682. 10.1371/journal.pone.0004682 19270752 PMC2650778

[2] Qian Z Shou-Yu G Feng-Xia Z Peng Y Wen-Jian S Jian-Liang L. Molecular characteristics of H9N2 influenza viruses isolated from farmed raccoon dogs and arctic foxes in China. Res Vet Sci. (2021) 135:542–6. 10.1016/j.rvsc.2020.11.006 33223121

[3] Kameo Y Nagao Y Nishio Y Shimoda H Nakano H Suzuki K. Epizootic canine distemper virus infection among wild mammals. Vet Microbiol. (2012) 154:222–9. 10.1016/j.vetmic.2011.07.006 21840141

[4] Jia-Yu Y Qian Z Fei-Fei D Chuan-Jie T Hui P Yuan-Yuan S. Emergence of novel canine parvovirus type 2 and its pathogenesis in raccoon dogs. Vet Microbiol. (2018) 216:7–12. 10.1016/j.vetmic.2018.01.016 29519528

[5] Shao X-Q Wen Y-J Ba H-X Zhang X-T Yue Z-G Wang K-J. Novel amdoparvovirus infecting farmed raccoon dogs and arctic foxes. Emerg Infect Dis. (2014) 20:2085–8. 10.3201/eid2012.140289 25417672 PMC4257837

[6] Song T Hao J Zhang R Tang M Li W Hui W. First detection and phylogenetic analysis of porcine circovirus type 2 in raccoon dogs. BMC Vet Res. (2019) 15:107. 10.1186/s12917-019-1856-2 30961660 PMC6454600

[7] Li J Cui L Deng X Yu X Zhang Z Yang Z. Canine bufavirus in faeces and plasma of dogs with diarrhoea, China. Emerg Microbes Infect. (2019) 8:245–7. 10.1080/22221751.2018.1563457 30866778 PMC6455112

[8] Fahsbender E Altan E Seguin MA Young P Estrada M Leutenegger C. Chapparvovirus DNA found in 4% of dogs with diarrhea. Viruses. (2019) 11:398. 10.3390/v11050398 31035625 PMC6563200

[9] Moreno PS Wagner J Kirkwood CD Gilkerson JR Mansfield CS. Characterization of the fecal virome in dogs with chronic enteropathy. Vet Microbiol. (2018) 221:38–43. 10.1016/j.vetmic.2018.05.020 29981706

[10] Yang S Liu Z Wang Y Li W Fu X Lin Y. A novel rodent Chapparvovirus in feces of wild rats. Virol J. (2016) 13:133. 10.1186/s12985-016-0589-0 27473724 PMC4966819

[11] Luo R Liu B Xie Y Li Z Huang W Yuan J. SOAPdenovo2: an empirically improved memory-efficient short-read *de novo* assembler. Gigascience. (2012) 1:18. 10.1186/2047-217X-1-18 23587118 PMC3626529

[12] Aiyar A. The use of CLUSTAL W and CLUSTAL X for multiple sequence alignment. Methods Mol Biol. (2000) 132:221–41. 10.1385/1-59259-192-2:221 10547838

[13] Kumar S Stecher G Li M Knyaz C Tamura K. MEGA X: molecular evolutionary genetics analysis across computing platforms. Mol Biol Evol. (2018) 35:1547–49. 10.1093/molbev/msy096 29722887 PMC5967553

[14] Cotmore SF Tattersall P. Parvoviruses: small does not mean simple. Annu Rev Virol. (2014) 1:517–37. 10.1146/annurev-virology-031413-085444 26958732

[15] Brown BA Maher K Flemister MR Naraghi-Arani P Uddin M Oberste MS. Resolving ambiguities in genetic typing of human enterovirus species C clinical isolates and identification of enterovirus 96, 99 and 102. J Gen Virol. (2009) 90:1713–23. 10.1099/vir.0.008540-0 19264596

[16] Kamar N Izopet J Pavio N Aggarwal R Labrique A Wedemeyer H. Hepatitis E virus infection. Nat Rev Dis Prim. (2017) 3:17086. 10.1038/nrdp.2017.86 29154369

[17] Haqshenas G Shivaprasad HL Woolcock PR Read DH Meng XJ. Genetic identification and characterization of a novel virus related to human hepatitis E virus from chickens with hepatitis-splenomegaly syndrome in the United States. J Gen Virol. (2001) 82:2449–62. 10.1099/0022-1317-82-10-2449 11562538

[18] Shi M Lin X-D Tian J-H Chen L-J Chen X Li C-X. Redefining the invertebrate RNA virosphere. Nature. (2016) 540:539–43. 10.1038/nature20167 27880757

[19] Williams SH Che X Garcia JA Klena JD Lee B Muller D. Viral diversity of house mice in New York City. MBio. (2018) 9:e01354-17. 10.1128/mBio.01354-17 PMC5904411 29666290

[20] Stainton D Martin DP Muhire BM Lolohea S Halafihi M Lepoint P. The global distribution of Banana bunchy top virus reveals little evidence for frequent recent, human-mediated long distance dispersal events. Virus Evol. (2015) 1:vev009. 10.1093/ve/vev009 27774281 PMC5014477

[21] Nagasaki K Tomaru Y Takao Y Nishida K Shirai Y Suzuki H. Previously unknown virus infects marine diatom. Appl Environ Microbiol. (2005) 71:3528–35. 10.1128/AEM.71.7.3528-3535.2005 16000758 PMC1169059

[22] Rosario K Breitbart M Harrach B Segalés J Delwart E Biagini P. Revisiting the taxonomy of the family Circoviridae: establishment of the genus Cyclovirus and removal of the genus Gyrovirus. Arch Virol. (2017) 162:1447–63. 10.1007/s00705-017-3247-y 28155197

[23] Varsani A Krupovic M. Smacoviridae: a new family of animal-associated single-stranded DNA viruses. Arch Virol. (2018) 163:2005–15. 10.1007/s00705-018-3820-z 29572596

[24] Huang Q Luo Y Cheng F Best SM Bloom ME Qiu J. Molecular characterization of the small nonstructural proteins of parvovirus Aleutian mink disease virus (AMDV) during infection. Virology. (2014) 452–3:23–31. 10.1016/j.virol.2014.01.005 24606679 PMC3955004

[25] Gorham JR Henson JB Crawford TB Padgett GA. The epizootiology of aleutian disease. Front Biol. (1976) 44:135–58. 182558

[26] Virtanen J Smura T Aaltonen K Moisander-Jylhä A-M Knuuttila A Vapalahti O. Co-circulation of highly diverse Aleutian mink disease virus strains in Finland. J Gen Virol. (2019) 100:227–36. 10.1099/jgv.0.001187 30526739

[27] Alex CE Kubiski S V Li L Sadeghi M Wack RF McCarthy MA. Amdoparvovirus Infection in Red Pandas(Ailurus fulgens). Vet Pathol. (2018) 55:552–61. 10.1177/0300985818758470 29433401

[28] Woo PCY Lau SKP Choi GKY Huang Y Teng JLL Tsoi H-W. Natural occurrence and characterization of two internal ribosome entry site elements in a novel virus, canine picodicistrovirus, in the picornavirus-like superfamily. J Virol. (2012) 86:2797–808. 10.1128/JVI.05481-11 22205729 PMC3302267

[29] Reuter G Boros Á Földvári G Szekeres S Mátics R Kapusinszky B. Dicipivirus (family Picornaviridae) in wild Northern white-breasted hedgehog (Erinaceus roumanicus). Arch Virol. (2018) 163:175–81. 10.1007/s00705-017-3565-0 28940090

[30] Rossmann MG He Y Kuhn RJ. Picornavirus-receptor interactions. Trends Microbiol. (2002) 10:324–31. 10.1016/S0966-842X(02)02383-1 12110211

[31] Lin J-Y Chen T-C Weng K-F Chang S-C Chen L-L Shih S-R. Viral and host proteins involved in picornavirus life cycle. J Biomed Sci. (2009) 16:103. 10.1186/1423-0127-16-103 19925687 PMC2785775

[32] Lee K-M Chen C-J Shih S-R. Regulation mechanisms of viral IRES-driven translation. Trends Microbiol. (2017) 25:546–61. 10.1016/j.tim.2017.01.010 28242053

[33] Van Nguyen D Harvala H Ngole EM Delaporte E Woolhouse MEJ Peeters M. High rates of infection with novel enterovirus variants in wild populations of mandrills and other old world monkey species. J Virol. (2014) 88:5967–76. 10.1128/JVI.00088-14 24623420 PMC4093852

[34] Gough RE Collins MS Borland E Keymer LF. Astrovirus-like particles associated with hepatitis in ducklings. Vet Rec. (1984) 114:279. 10.1136/vr.114.11.279-a 6424324

[35] Madeley CR Cosgrove BP. 28 nm particles in fæces in infantile gastroenteritis. Lancet. (1975) 2:451–2. 10.1016/S0140-6736(75)90858-2 51251

[36] Chen XU He Y Li W Kalim U Xiao Y Yang J. Identification and characterization of a novel recombinant porcine astrovirus from pigs in Anhui, China. Polish J Microbiol. (2020) 69:471–8. 10.33073/pjm-2020-051 33574875 PMC7812366

