# Supplementary figures and images for: Viral Metagenomics Reveals Diverse Viruses in the Feces Samples of Raccoon Dogs
Source: Front Vet Sci. 2021 Jul 12;8:693564. doi: 10.3389/fvets.2021.693564 (PMC8311183; doi:10.3389/fvets.2021.693564)

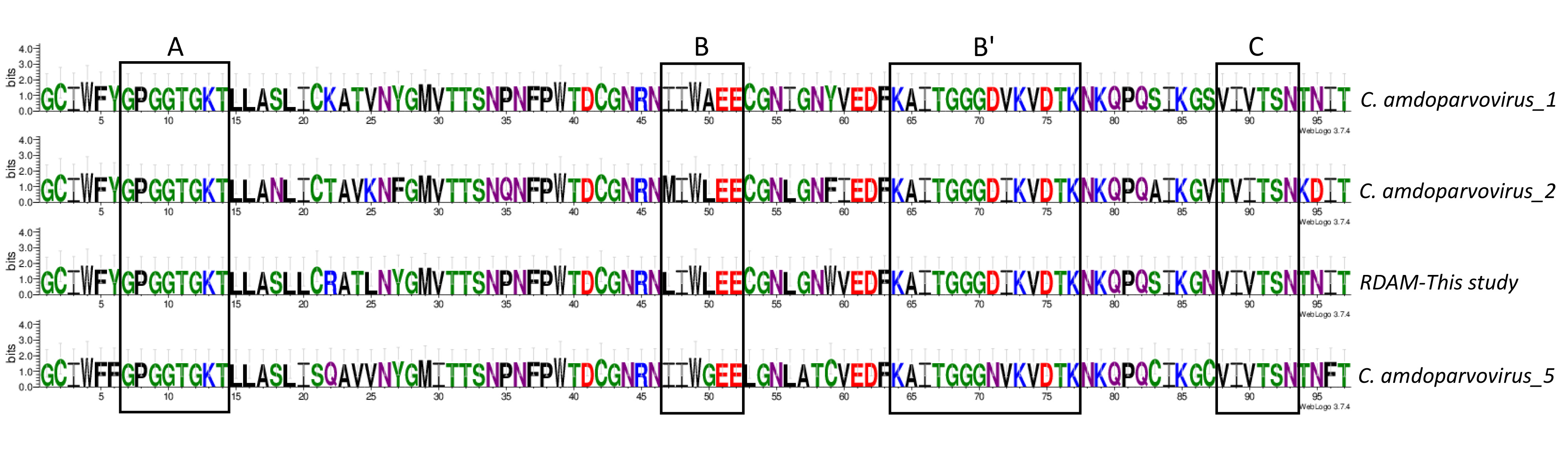

Supplement: Supplementary Figure 1 — Comparison of amino acid sequences of helicase motifs in members of the genus Amdoparvovirus. A sequence logo for each species was generated with Weblogo 3. Four walker domains were marked with square boxes. [file Image_1.JPEG]

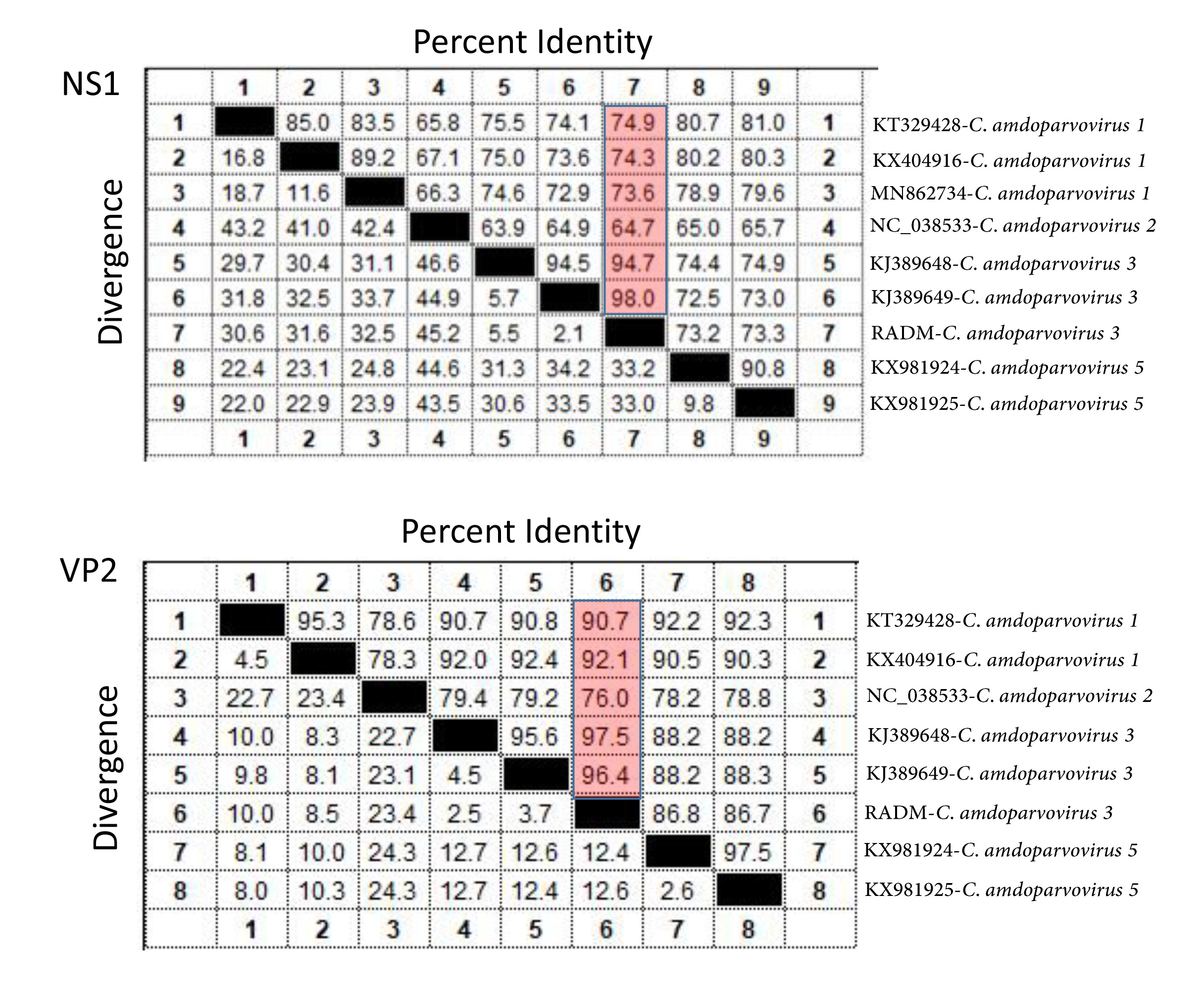

Supplement: Supplementary Figure 2 — Pairwise comparison of RADM with the reference strains of Canine amdoparvoviruses. (A) Based on the NS1. (B) Based on the VP2. The percent identity between RADM and other strains was marked with red. [file Image_2.JPEG]
