# Supplementary material for: Viral Metagenomics Reveals Diverse Viruses in the Feces Samples of Raccoon Dogs
Source: Front Vet Sci. 2021 Jul 12;8:693564. doi: 10.3389/fvets.2021.693564 (PMC8311183; doi:10.3389/fvets.2021.693564)
Supplement: Supplementary Table 1 — The Summary of library information of raccoon dogs. [file Table_1.DOCX]

Table S1. The summary of library information of raccoon dogs

| Library ID | Sample type | No. of sample | Healthy status | Total no. of raw reads | No. of viral reads with E value<10^-5^ |
| --- | --- | --- | --- | --- | --- |
| 1 | Feces | 10 | Normal | 2,163,948 | 37,079 |
| 2 | Feces | 10 | Normal | 3,335,580 | 46,871 |
| 3 | Feces | 10 | Normal | 2,790,968 | 49,284 |
