# Supplementary material for: Viral Metagenomics Reveals Diverse Viruses in the Feces Samples of Raccoon Dogs
Source: Front Vet Sci. 2021 Jul 12;8:693564. doi: 10.3389/fvets.2021.693564 (PMC8311183; doi:10.3389/fvets.2021.693564)
Supplement: Supplementary Table 2 — The Sequence numbers of animal virus in different libraries of this study. [file Table_2.DOCX]

Table S2. The sequence numbers of animal virus in different libraries of this study.

| Virus Families | No. of viral reads with E value<10^-5^ | | |
| --- | --- | --- | --- |
|  | Library1 | Library2 | Library3 |
| Adenoviridae | 14 | 15 | 5 |
| Anelloviridae | 11 | 11 | 20 |
| Arenaviridae | 0 | 2 | 3 |
| Arteriviridae | 3 | 2 | 0 |
| Asfarviridae | 7 | 3 | 2 |
| Astroviridae | 8 | 99 | 29 |
| Caliciviridae | 12 | 14 | 18 |
| Circoviridae | 868 | 1290 | 703 |
| Coronaviridae | 17 | 23 | 13 |
| Dicistroviridae | 19 | 32 | 156 |
| Flaviviridae | 18 | 5 | 12 |
| Geminiviridae | 20 | 9 | 17 |
| Herpesviridae | 19 | 27 | 47 |
| Iridoviridae | 42 | 48 | 56 |
| Papillomaviridae | 8 | 9 | 3 |
| Paramyxoviridae | 4 | 1 | 5 |
| Parvoviridae | 459 | 262 | 547 |
| Picobirnaviridae | 147 | 550 | 170 |
| Picornaviridae | 505 | 3531 | 998 |
| Poxviridae | 5 | 2 | 18 |
| Reoviridae | 8 | 14 | 9 |
| Retroviridae | 13 | 5 | 8 |
